# Predictors for COVID-19 Complete Remission with HRCT Pattern Evolution: A Monocentric, Prospective Study

**DOI:** 10.3390/diagnostics12061397

**Published:** 2022-06-05

**Authors:** Diana Manolescu, Bogdan Timar, Felix Bratosin, Ovidiu Rosca, Cosmin Citu, Cristian Oancea

**Affiliations:** 1 Department of Radiology, “Victor Babes” University of Medicine and Pharmacy Timisoara, Eftimie Murgu Square 2, 300041 Timisoara, Romania; dmanolescu@umft.ro; 2 Center for Research and Innovation in Precision Medicine of Respiratory Diseases, “Victor Babes” University of Medicine and Pharmacy Timisoara, Eftimie Murgu Square 2, 300041 Timisoara, Romania; oancea@umft.ro; 3 Department of Internal Medicine II, Division of Diabetes, Nutrition and Metabolic Diseases, “Victor Babes” University of Medicine and Pharmacy Timisoara, Eftimie Murgu Square 2, 300041 Timisoara, Romania; 4 Center for Molecular Research in Nephrology and Vascular Disease, Faculty of Medicine, “Victor Babes” University of Medicine and Pharmacy Timisoara, Eftimie Murgu Square 2, 300041 Timisoara, Romania; 5 Methodological and Infectious Diseases Research Center, Department of Infectious Diseases, “Victor Babes” University of Medicine and Pharmacy Timisoara, Eftimie Murgu Square 2, 300041 Timisoara, Romania; felix.bratosin7@gmail.com (F.B.); ovidiu.rosca@umft.ro (O.R.); 6 Department of Obstetrics and Gynecology, “Victor Babes” University of Medicine and Pharmacy Timisoara, 300041 Timisoara, Romania; citu.ioan@umft.ro

**Keywords:** COVID-19, SARS-CoV-2 infection, imaging studies, HRCT, disease remission, prediction model

## Abstract

There are growing concerns that some COVID-19 survivors may acquire fibrosis and other irreversible lung abnormalities. The purpose of this prospective study was to assess the rate and predictors of complete resolution of COVID-19 pneumonia by pursuing a hypothetical relation between time and imaging pattern evolution using HRCT findings. A monocentric prospective cohort study with a consecutive-case enrolment design was implemented during a five-month period, having a total of 683 post-COVID patients eligible for inclusion and 635 evaluations with complete follow-up for chest HRCT. The target for post-COVID evaluations consisted of performing HRCT 90 days after a confirmed SARS-CoV-2 infection. The studied patients had an average age of 54 years, ranging between 18 and 85 years old, and an average duration from the first symptoms until HRCT was performed of 74 days. At the post-COVID follow-up, 25.8% had a complete imagistic remission. The most common appearance with HRCT was “ground glass” in 86.6% in patients with persistent COVID-19, followed by reticulations, present in 78.8%, and respectively pleural thickening in 41.2% of cases. The mean total HRCT scores were statistically significantly higher in patients older than 65 years (10.6 ± 6.0) compared to the 40–65 group (6.1 ± 6.1) and the 18–40 age group (2.7 ± 4.8) (*p* < 0.001). Chest HRCT is a “time window” in documenting temporal persistent radiologic features of lung injury 90 days after SARS-CoV-2 infection, determining the pathologic basis of so-called “long COVID”. The complete remission was associated with a significantly higher average follow-up period and a significantly lower average patient age. Persistent HRCT features of ground glass, reticulation, and pleural thickening are associated with a higher total CT score and older age.

## 1. Introduction

The coronavirus disease 2019 or COVID-19 is caused by the novel coronavirus known as the severe acute respiratory syndrome coronavirus 2 (SARS-CoV-2) [1]. It has become a worldwide outbreak since the first report in December 2019 and was declared a pandemic by the World Health Organization on 11 March 2020 [2]. With more than 300 million cases, this disease carries the burden of a global impact with more than 5 million deaths [3].

Previous pathology studies highlighted that SARS-CoV-2 viral infection could cause multiple organ and tissue injuries with major pulmonary impact [4,5]. Acute fibrous and organizing pneumonia with an extensive intra-alveolar fibrin deposition, rather than hyaline membranes of the classic diffuse alveolar damage, is the hallmark of lung injury in severe SARS-CoV-2 infection [6,7]. There is a general concern if these “fibrotic-like” lung changes are truly an irreversible disease process in a post-ARDS setting [8], as the presence of alveolar septal fibrosis results in a substantial increase in diffuse alveolar damage related to a greater density of mature type-I collagen and immature type-III collagen [9]. On the contrary, other studies suggest that some of these findings will resolve over time and are therefore not truly fibrotic [10]. This could be related to the “gray area” of immature fibrosis and fibroblastic changes that can remodel with time, and therefore fibrotic patterns on initial CT continue to regress in the longer-term follow-up CT [11].

Nevertheless, due to the novelty of this pathologic entity, further long-term investigations are needed to obtain a comprehensive knowledge of COVID-19 outcomes and pulmonary sequels. The aim of this prospective study was to assess the rate and the predictors of complete resolution of COVID-19 pneumonia in a large cohort of survived patients and to pursue a hypothetical relation between time and imaging pattern evolution in gradual follow-up high-resolution computed tomography (HRCT) extended up to 120 days.

## 2. Materials and Methods

### 2.1. Patients and Study Design

In this prospective cohort study that took place between 15 November 2020 and 10 May 2021, a consecutive-case enrollment scenario was implemented, having a total of 683 post-COVID patients eligible for inclusion. At the end of the study, a total of 635 evaluations were performed. As inclusion criteria, all adult individuals older than 18 years with SARS-CoV-2 infection necessitating inpatient medical care were considered. All patients were admitted for COVID-19 in our hospital and had a positive real-time polymerase chain reaction test (RT-PCR) for SARS-CoV-2 viral RNA from oropharyngeal and nasal swabs at the moment of admission. Another inclusion criterion was the pulmonary evaluation by imaging studies at the time of admission and acceptance of HRCT at the term follow-up period.

The hospital admission criteria for COVID-19 patients involved symptomatic cases requesting treatment and oxygen supplementation based on the following severity criteria: A low-grade fever without pneumonia was characterized as a moderate type of COVID-19 infection. The moderate type was characterized by the presence of fever and non-severe pneumonia symptoms without the requirement for oxygen therapy. The severe cases were defined by respiratory failure requiring mechanical ventilation, a respiratory rate greater than 30/min, a hemoglobin oxygen saturation of 90% as measured by pulse oximetry (SpO2), coagulation disorders, organ failure requiring ICU admission, and ground-glass opacities involving more than 50% of the lungs on chest X-ray or CT scan. All patients brought to our clinic with COVID-19 infection were treated with a typical combination of antivirals, steroids, antibiotic prophylaxis for subsequent bacterial pneumonia, and anticoagulation, with changes made according to the patient profile.

### 2.2. Study Variables and Scores

The variables considered for evaluation in the current study comprised patient background characteristics (age, gender, place of residence), COVID-19 features (duration from symptoms onset until a positive RT-PCR test result, confirmation of complete remission), imaging studies (presence of ground-glass opacities, crazy-paving pattern, condensing features, trabeculations, and reticular pattern, bronchiectasis, air-filled cysts, pulmonary tractions, pleural thickening), and lobular involvement (right upper lobe, right middle lobe, right lower lobe, left upper lobe, lingula, left lower lobe). In the study cohort, patients with abnormal findings on HRCT follow-up evaluation are considered as a persistent group, while those who show complete disease remission on imaging studies have no signs of lung injury and are considered as the non-persistent group. The target for post-COVID evaluations consisted of performing HRCT 90 days after a confirmed SARS-CoV-2 infection. A six-lobe evaluation was considered, with the left lingula being considered a lobe of the left lung. The maximum CT score was 30, calculated on a 0 to 5 scale, using a previously validated scoring system [12]. The severity score was considered mild when lower than 10, moderate between 10 and 20, and severe if higher than 20 (Table 1).

### 2.3. Ethics

The Ethics Committee at the “Victor Babes” University of Medicine and Pharmacy in Timisoara, Romania accepted the study procedure. The study followed the Declaration of Helsinki guidelines and was approved by the Ethics Committee of the “Victor Babes” Clinical Hospital for Infectious Diseases and Pulmonology in Timisoara, which operates in accordance with Article 167 of Law No. 95/2006, Art. 28, Chapter VIII of Order 904/2006; with the European Union’s Good Clinical Practice Directives 2005/28/EC; and with the International Conference on Harmonization of Technical Requirements for Registration of Pharmaceuticals.

### 2.4. Statistical Analysis

Data were collected and analyzed using SPSS Statistical Software (IBM Corp. Chicago, IL, USA). The results are presented as average ± standard deviation for numerical variables with Gaussian distribution, median and interquartile range (IQR) for continuous variables without normal distribution, respectively, as absolute frequencies and percentages from the sub-groups total for categorical variables. The analyzed variable’s distribution was evaluated with Kolmogorov and Smirnoff’s test and the equality of the variable’s variations using Levene’s test. The Kruskal–Wallis test was used to compare non-Gaussian variables, while the normally distributed variables were compared using the ANOVA test. A receiver-operating characteristics (ROC) analysis was performed to analyze the time elapsed from the first positive test as a predictor for complete remission of COVID-19 pathologic imagistic signs.

## 3. Results

At the end of the follow-up period, a total of 48 patients were excluded from the study for missed follow-up or death from COVID-19 complications or associated comorbidities. In the final analysis, there were 635 patients, out of which 471 were considered persistent cases when the clinical symptoms and imaging studies did not show complete disease remission at 90 days following a negative RT-PCR test for SARS-CoV-2. In the study cohort, 53.4% of the patients were men, respectively 46.6% were women. The studied patients had an average age of 53.9 ± 13.4 years, ranging between 18 and 85 years old, and an average duration from the first symptoms until an HRCT study was performed of 73.7 ± 47.3 days, ranging between 15 and 65 days. The majority of patients (68.7%) had their place of residence in urban areas, while the remaining 31.3% came from rural regions.

### 3.1. Imaging Studies

**Figure 1:**
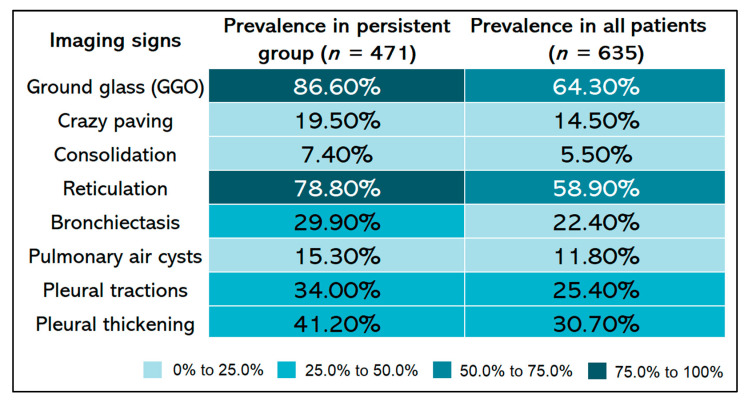
The prevalence of persistent post-COVID imaging signs using HRCT at 90 days follow-up.

**Figure 2:**
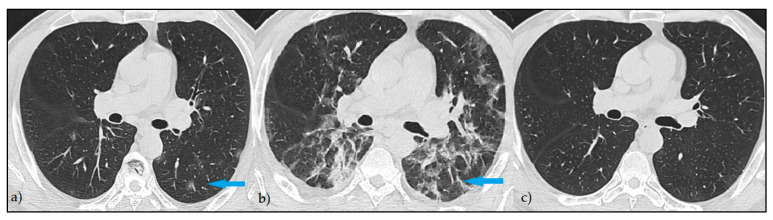
HRCT pattern of complete remission—Axial HRCT images: (**a**) minimal focal areas of GGO at the onset of the symptoms (blue arrow); (**b**) consolidation with a reticular pattern at 14 days from diagnostic (blue arrow); (**c**) complete remission of the lesions at 3 months.

**Figure 3:**
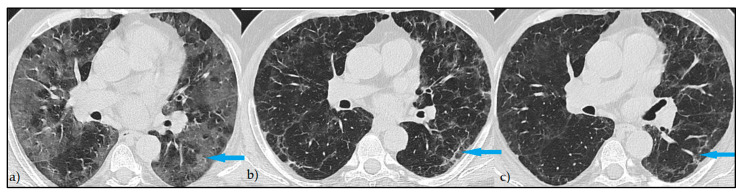
Persistent HRCT findings—Axial HRCT images: (**a**) extensive areas of GGO at the onset of the symptoms (blue arrow); (**b**) reticular pattern at 14 days from diagnostic (blue arrow); (**c**) persistent reticulation at 3 months with slightly decreased in attenuation (blue arrow).

**Figure 4:**
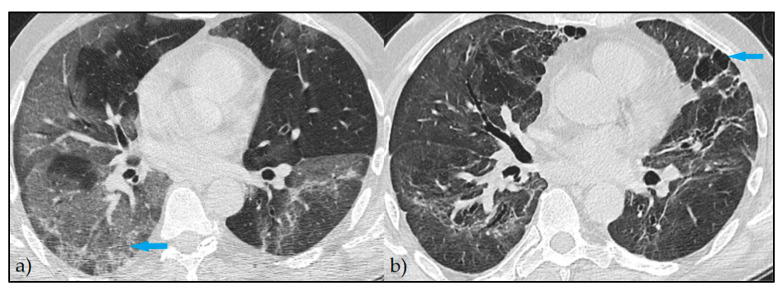
Persistent HRCT findings—Axial HRCT images: (**a**) extensive areas of GGO at the onset of the symptoms (blue arrow); (**b**) persistent areas of fine GGO with bronchiectasis, pleural traction, and pleural thickening at 3 months evaluation (blue arrow).

At the post-COVID follow-up, 471 (74.1%) patients had persistent imaging signs, as described in Figure 1, while 164 patients (25.8%) had a complete imagistic remission, as represented by Figure 2. The complete remission was associated with a significantly higher follow-up period (84.7 ± 57.8 vs. 70.1 ± 42.6 days; *p* = 0.003) and a significantly lower age (44.4 ± 11.6 vs. 57.2 ± 12.4 years; *p* < 0.001). In the group of patients without complete remission, the median number of imagistic signs was 3, with an interquartile range of 2 (minimum 1, maximum eight imagistic signs). A higher number of imagistic signs was associated with a higher total score (Kendall’s tau = 0.509; *p* < 0.001) and a higher age (Kendall’s tau = 0.232; *p* < 0.001). No significant association was observed between the number of imagistic signs and the elapsed duration from the first positive COVID PCR test (Kendall’s tau = -0.011; *p* = 0.743). The prevalence of persistent post-COVID imaging signs is presented in Figure 1. It was observed that the most common lung appearance with HRCT was “ground glass” for a total of 86.6% in patients with persistent COVID-19 (Figure 3), followed by reticulations, present in 78.8%. The third most common imagistic feature was the existence of pleural thickening (41.2%), as seen in Figure 4, while the least common feature was represented by consolidation appearance in only 35 patients (7.4%).

### 3.2. Time Intervals and Age Group Analysis

The time interval analysis presented in Table 2 identified a majority of 46.02% of patients enrolled in the current study being evaluated between 30 and 60 days with HRCT after the first positive COVID-19 test and hospital admission. A total of 21.78% of the patients were evaluated between 60 and 90 days, while only 5.68% benefited from HRCT at a period shorter than 30 days from the positive PCR test.

**Figure 5:**
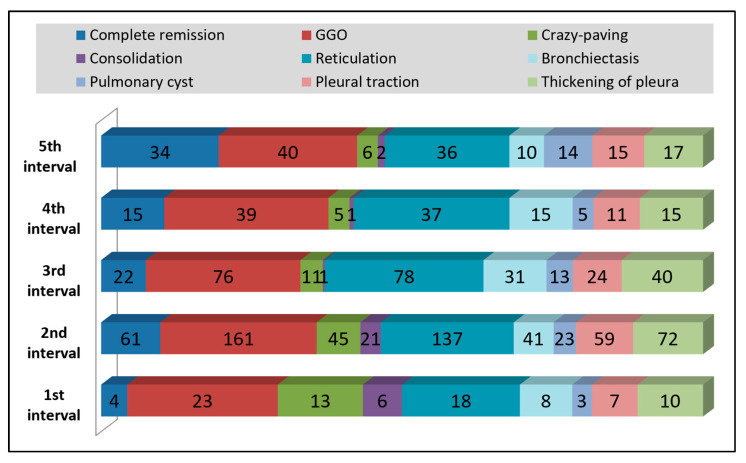
Evolution of HRCT findings by frequency in all patients included in the study.

Most of the pulmonary imaging findings within 30 days from hospital admission were ground-glass opacities in 23 patients, followed by 18 patients with reticulations (Figure 5). Between 30 and 60 days from admission, there were already 61 patients with complete remission of pathologic lung findings on HRCT, although 161 still manifested GGO and 137 reticulations. Pleural thickenings were the third most common observation in this group, with manifestations in 72 patients. Between 60 and 90 days from admission, it was discovered that bronchiectasis and pleural thickenings became more frequent, although GGO and reticulations were the most prevalent in 76 and, respectively, 78 patients.

A comparison of imagistic findings stratified by time intervals elapsed from the first positive COVID-19 test until HRCT, presented in Table 3, identified statistically significant differences in proportions of ground-glass opacities and crazy paving patterns, condensing features, and cases of full remission. The average total HRCT score between the five intervals of study showed statistically significant differences between groups (*p*-value < 0.001), with pairwise comparisons identifying significant differences between the first interval and all other four intervals. A similar comparison stratified by patients’ age groups, described in Table 4, observed that all lung injury characteristics were statistically significantly different by the proportion between the three age groups. The mean total HRCT scores were statistically significantly higher in patients older than 65 years (10.6 ± 6.0) compared to the 40–65 group (6.1 ± 6.1) and the 18–40 age group (2.7 ± 4.8) (*p* < 0.001).

**Figure 6:**
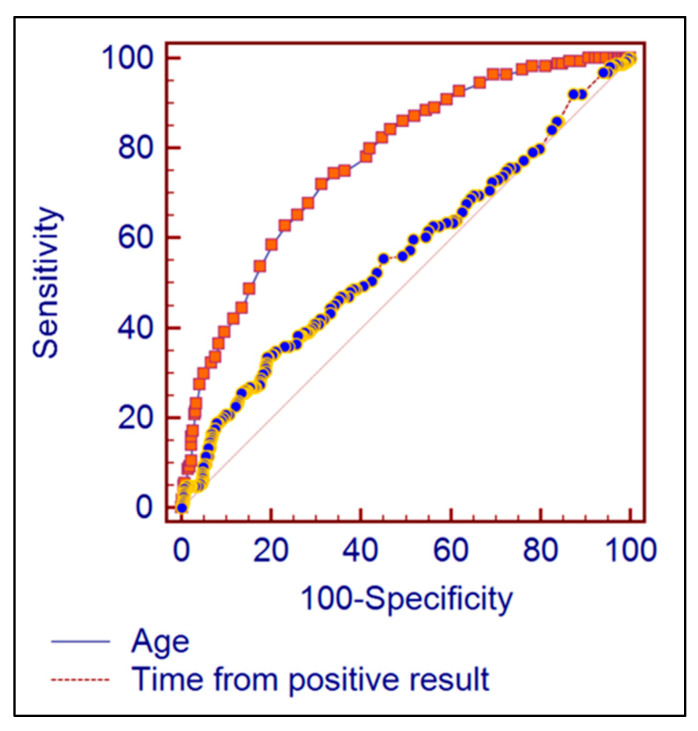
Comparison of AUROC between patient’s age and time from first positive COVID-19 test.

At the receiver-operating characteristic analysis, the area under the ROC curve for using the time from positive SARS-CoV-2 RT-PCR result as a predictor for complete remission was 0.564 (95% CI = 0.524 to 0.603), being statistically significantly different from the non-discriminating AUROC = 0.5 (*p* = 0.0183). The best cut-off prediction value for complete remission of imaging signs was 96 days elapsed from the first positive RT-PCR COVID-19 test, this Youden index having a 33.54% sensitivity and 80.89% specificity. The patient’s age proved to be a valid predictor for the complete remission of imagistic signs, with the best discrimination Youden index being an age of lower than 50 years having the area under the ROC equal to 0.772 (95% CI = 0.738 to 0.805), being significantly different from the non-discriminating AUROC value (*p* < 0.001). The age lower than 50 years had a sensitivity of 71.95%, respectively a specificity of 68.58% for predicting a complete remission (Figure 6). The patient’s age was a stronger predictor of complete remission compared to the time elapsed from the first positive COVID-19 test (AUROC = 0.772 vs. 0.564; *p* < 0.001).

## 4. Discussion

### 4.1. Current Observations and Literature Findings

The current study demonstrates through the help of HRCT how patients with a short duration from symptoms onset until a positive RT-PCR test result that RT-PCR are likely to manifest persistent radiologic features of lung injury 90 days after SARS-CoV-2 infection, which determines the pathologic basis of so-called “long COVID”. With an important role in the initial diagnosis and also in the follow-up of patients with COVID-19 pneumonia, chest HRCT is “a time window” in documenting temporal radiographic changes. As the present study observed radiologic pulmonary changes with the purpose of predicting the outcomes of lung damage after SARS-CoV-2 infection, several other studies commenced at the very beginning of the COVID-19 pandemic should be credited for the initiative of standardizing an HRCT scoring system.

Additionally, quantitative chest HRCT and semiquantitative lung severity scores analyses were used in research studying COVID-19 patients. To measure lung lesions, semiquantitative evaluation approaches such as lobar and segmental-based CT scores, opacity-weighted scores, and quantitative assessment methods such as lesion volume quantification were used. Inter-rater agreement was high for all four evaluation techniques. At the group level, lesion burden was consistently seen to be considerably greater in severe type patients than in common type patients when four evaluation techniques were used. It was established that both semiquantitative and quantitative approaches had great repeatability when evaluating inflammatory lesions and are capable of distinguishing between patients with the common type and those with the severe type [13,14]. The severity of the initial disease was associated with residual lung changes in 42% of discharged COVID-19 patients included in a mid-term follow-up study [15]. A recent study demonstrated that more than one-third of survivor patients with severe COVID-19 pneumonia presented fibrotic-like changes in the six-month follow-up HRCT, associated with older age and higher initial chest HRCT score [16].

Liu et al. [17] observed among the first hospitalized patients who survived a severe SARS-CoV-2 infection that the radiographic changes over time are an excellent predictor of viral clearance. According to the authors, HRCT scans revealed several imaging features shared by our patients as well, including ground-glass opacity, consolidation, air bronchogram, nodular opacities, and pleural effusion. The HRCT peak scores throughout the illness course were substantially greater in COVID-19 patients with severe pneumonia than in those with mild COVID-19, with a statistically significant increase in consolidation and air bronchogram. The median number of days before peak HRCT scores were obtained was 12 in pneumonia patients and 14 in severe pneumonia patients, respectively. Another study [17] described for the majority of patients that CT scans revealed bilateral, multifocal lung lesions involving several lobes and a diffuse distribution, with progressive alterations in comparison to the baseline CT score and a CT score peaking at 12 days and a nadir at 26 days. The remaining 25% of patients showed no improvement in comparison to their baseline CT score, and the lowest CT score was achieved at 23 days [18].

Previous research has revealed comparable radiological features to the current study at different stages in the acute course of COVID-19 that ranged from mild to severe cases. Yasin et al. observed that two-thirds of the patients involved had abnormal radiologic pulmonary findings of bilateral lung injury [19]. More than eighty percent of the patients showed consolidation opacities at follow-up, followed by a third of patients presenting features of reticular interstitial thickening, and, respectively, another third from the study cohort had ground-glass opacities (GGO). Similarly, a study by Ozturk et al. found that multilobar, peripherally dispersed mixed ground-glass opacities and consolidation, crazy paving, and a higher overall CT severity score were strongly linked with severe COVID-19 on chest computed tomography images [20].

A longitudinal study commenced at the beginning of the COVID-19 pandemic found that approximately 94% of the studied patients had residual disease on CT scans at discharge, with the most common persistent pattern being ground-glass opacities [21]. In these patients, however, the peak CT scores were predominantly at five days after hospital admission, in contrast with our findings or other research previously discussed where the scores peaked after ten days of hospital admission. It is hypothesized that such differences can occur due to the high variability of the SARS-CoV-2 virus and different strains circulating at different times when studies were ongoing. Although GGO is very common at discharge, other studies reported that GGO opacities could be absorbed completely at three weeks in 53% of the discharged patients [22]. Additionally, it was previously observed how radiologic findings correspond to clinical signs of long COVID. Survivors with dyspnea had a significantly higher lesion volume on the CT scan, while the absorption of lesions continued to persist 6 months following hospital discharge [23]. Besides persistent respiratory complaints, patients with long COVID also showed continuously significantly higher values of c-reactive protein, fibrinogen, urea, and creatinine associated with abnormal imaging findings [24,25].

### 4.2. Limitations and Future Perspectives

Despite providing an important piece of evidence about patients with persistent lung injury after COVID-19 from a large group of patients, the current study has several limitations worth mentioning. The confusion bias with confounding factors should be considered, as the patient selection was not restricted by pre-existing lung disease, and in some cases, based on the radiologic findings it was impossible to determine if their onset was caused by SARS-CoV-2 infection alone or other unknown past medical history. In the same manner, it was unknown how many of the patients already had a previous SARS-CoV-2 infection that was left unnoticed and created the proper environment for irreversible lung damage at reinfection. It was also unknown which viral strains were responsible for infection since viral sequencing was not performed. Therefore, the findings presented in the current study can change with different SARS-CoV-2 variants.

The current study raises future perspectives in understanding the pulmonary involvement of long COVID syndrome. It promotes a further follow-up analysis of patients with persistent radiologic findings to observe whether a slow but significant remission is likely to occur, or irreversible fibrosis happens. Another key question is to determine which particular pulmonary features are early predictors for irreversible lung damage.

## 5. Conclusions

HRCT imaging studies should be utilized more often in the setting of the COVID-19 pandemic, since they prove to have an important role in assessing disease severity at hospital admission and following prognosis. HRCT chest exams performed throughout the course of illness help in patient treatment, and provide a stronger association of the patient clinical presentation with their radiological features that might benefit in further decreasing the SARS-CoV-2 infection-related complications and deaths. With the increasing number of patients complaining of persistent symptoms after SARS-CoV-2 viral clearance, HRCT performed at 90 days after COVID-19 can help diagnose so-called “long COVID” and determine a management plan.

## Figures and Tables

**Table 1:** Computed Tomography severity scores.

Scores	Categories
**Lung lobe score**	
0	No lobar involvement
1	<5%
2	5–25%
3	25–50%
4	50–75%
5	>75%
**CT Severity Score**	
<10	Mild
10–20	Moderate
>20	Severe

**Table 2:** Distribution of patients based on time intervals elapsed from the first positive COVID-19 test.

Interval	Period	Frequency
1st Interval	<30 days	5.68%
2nd Interval	30–60 days	46.02%
3rd Interval	60–90 days	21.78%
4th Interval	90–120 days	10.98%
5th Interval	>120 days	15.53%

**Table 3:** Comparison of imagistic findings in the study cohort stratified by time intervals elapsed from the first positive COVID-19 test.

HRCT Findings	1st Interval (*n* = 33)	2nd Interval (*n* = 267)	3rd Interval (*n* = 154)	4th Interval (*n* = 76)	5th Interval (*n* = 105)	*p*-Value *
Complete remission (*n* = 164)	13 (7.9%)	59 (36.0%)	33 (20.1%)	20 (12.2%)	39 (23.8%)	0.014
Ground-glass opacities (*n* = 408)	47 (11.5%)	164 (40.2%)	101 (24.8%)	47 (11.5%)	49 (12.0%)	0.014
Crazy paving (*n* = 92)	21 (22.8%)	44 (47.8%)	17 (18.5%)	3 (3.3%)	7 (7.6%)	<0.001
Condensing (*n* = 35)	11 (31.4%)	20 (57.1%)	2 (5.7%)	1 (2.9%)	1 (2.9%)	<0.001
Trabeculation (*n* = 374)	35 (9.4%)	145 (38.8%)	100 (26.7%)	45 (12.0%)	49 (13.1%)	0.121
Bronchiectasis (*n* = 142)	14 (9.9%)	47 (33.1%)	43 (30.3%)	17 (12.0%)	21 (14.8%)	0.311
Pulmonary cysts (*n* = 75)	5 (6.7%)	29 (38.7%)	17 (22.7%)	7 (9.3%)	17 (22.7%)	0.390
Tractions (*n* = 161)	16 (9.9%)	65 (40.4%)	39 (24.2%)	18 (11.2%)	23 (14.3%)	0.984
Pleural thickening (*n* = 195)	18 (9.2%)	79 (40.5%)	52 (26.7%)	19 (9.7%)	27 (13.8%)	0.633
HRCT total score	10.5 ± 8.0	6.4 ± 6.0	7.2 ± 6.4	7.1 ± 6.7	4.9 ± 6.1	<0.001

* Data reported as *n* (%) and calculated using Chi-square test unless specified differently.

**Table 4:** Comparison of imagistic findings in the study cohort stratified by age group.

HRCT Findings	18–40 Years	40–65 Years	>65 Years	*p*-Value *
Complete remission (*n* = 164)	55 (33.5%)	103 (62.8%)	6 (3.7%)	<0.001
Ground-glass opacities (*n* = 408)	29 (7.1%)	245 (60.0%)	134 (32.8%)	<0.001
Crazy paving (*n* = 92)	3 (3.3%)	42 (45.7%)	47 (51.1%)	0.008
Condensing (*n* = 35)	2 (5.7%)	14 (40.0%)	19 (54.3%)	<0.001
Trabeculation (*n* = 374)	24 (6.4%)	229 (61.2%)	121 (32.4%)	<0.001
Bronchiectasis (*n* = 142)	5 (3.5%)	80 (56.3%)	57 (40.1%)	<0.001
Pulmonary cysts (*n* = 75)	0 (0.0%)	39 (52.0%)	36 (48.0%)	0.030
Tractions (*n* = 161)	10 (6.2%)	97 (60.2%)	54 (33.5%)	<0.001
Pleural thickening (*n* = 195)	11 (5.6%)	110 (56.4%)	74 (37.9%)	<0.001
HRCT total score	2.7 ± 4.8	6.1 ± 6.1	10.6 ± 6.0	<0.001

* Data reported as *n* (%) and calculated using the Chi-square test and Fisher’s exact test unless specified differently.

## Data Availability

Data available on request.
